# Infliximab versus cyclosporine for severe ulcerative colitis refractory to steroids: A protocol for systematic review and meta-analysis

**DOI:** 10.1097/MD.0000000000012657

**Published:** 2018-10-12

**Authors:** Dongjie Wu, Zhen Yang, Chen Zhao, Liang Yao

**Affiliations:** a School of Chinese Medicine, Hong Kong Baptist University, Hong Kong, PR China; b Graduate School, Zhejiang Chinese Medical University, Hangzhou, China.

**Keywords:** acute severe ulcerative colitis, long term, randomized controlled trials, steroid resistant, systematic review

## Abstract

**Background:**

Infliximab and cyclosporine are two main therapies in treating acute severe ulcerative colitis (ASUC), our objective is to assess the effectiveness and safety of cyclosporine (CSA) versus infliximab (IFX) as rescue agents in patients with steroid-refractory ulcerative colitis (UC).

**Methods:**

We will search three databases from inception to May 2018, and 19 studies are identified that infliximab and cyclosporine as a treatment in steroid-refractory UC patients. The primary outcome was short-term response to treatment. Secondary outcomes included the rates of colectomy at 3 months, 12 months, 36 months, adverse drug reactions and mortality in those who received rescue therapy.

**Results:**

This update review will provide a high quality synthesis of current evidence of two treatment for steroid-refractory ulcerative colitis. The definition of severe colitis is according to Truelove and Witts’ criteria.

**Conclusions:**

In the treatment of steroid-resistant ulcerative colitis with infliximab and cyclosporine, there is no difference between the two treatments on short-term and long-term results.

## 1 Introduction

Ulcerative colitis is the most common gastroenterology disease worldwide, and has a high incidence of morbidity in western countries,^[1]^ it affects approximately 2 million people in Europe.^[2]^ In the United States, about 593,000 cases of ulcerative colitis were estimated in 2009.^[3]^ The annual incidence rate in Europe is 24.3 per 100,000 people, and 6.3 per 100,000 person in Asia and the Middle East.^[4,5]^ Compared with Western countries, the incidence of ulcerative colitis is also increasing in developing countries.^[6,7]^ Due to the characteristic of ulcerative colitis, ulcerative colitis patients will increase their family burden, reduce in the ability to work and suffer from the drawback of the disease such as social stigma, difficulty with physical intimacy and restriction in career choices.^[5]^

Ulcerative colitis with severe acute exacerbations influences up to 25% of patients, either on the first-line treatment or later, and requires medical institutions confirmation for treatment with intravenous steroids.^[8]^ However, around 30% of these patients are impervious to steroid treatment and colectomy was the typical option is associated with short-term and long-term complications.^[9,10]^ In order to drop colectomy rates, several controlled trials have exhibited the viability of both cyclosporine, a calcine urine inhibitor, and infliximab, a hostile-to-tumor necrosis factor (TNF) monoclonal immune response, as second-line medicinal treatment.^[11]^ Infliximab and cyclosporine are similar effective medical therapies in treating intense serious ulcerative colitis, several trials have straightforwardly or in a roundabout way contrasted cyclosporine and infliximab in patients and steroid-recalcitrant ASUC. We still have not clear evaluation about their clinical effectiveness and safety.

An ongoing randomized control trial suggested that cyclosporine was not better than IFX in patients with steroid-refractory acute severe ulcerative colitis.^[11]^ The latest systematic review concluded that cyclosporine and infliximab had demonstrated similar short-term results as second-line therapies.^[12]^ This study included in 3 RCT studies published in 2016, and its outcome contained short-term response to treatment, the rates of colectomy at 3 months and 12 months, adverse drug reactions and mortality. But due to the publish time of inclusion experimental studies, yet long-term results for more than 36 months has not been clearly collected.^[12]^ Our review increases the number of studies and adds the long-term results of the original test report. According to the previous research, we collect clinical evidence for the effects of the infliximab and cyclosporine to provide evidence-based medicine for clinical guidance.

## 2 Methods

### 2.1 Registration

Protocol registration number in the PROSPERO registry of the University of York: (CRD42018094218). This systematic review protocol was reported using the Preferred Reporting Items for Systematic Reviews and Meta-Analyses (PRISMA-P) statement guidelines.^[13]^

### 2.2 Search methods for identifying the studies

#### 2.2.1 Electronic sources

The following databases will be searched from inception to date:

1. PubMed;
2. Embase;
3. The Cochrane Library (CENTRAL).

#### 2.2.2 Search strategy

The searches will be combined with the medical subject headings (Mesh) and keywords of “Infliximab OR Cyclosporine” AND “Ulcerative Colitis.” We will determine the search strategy based on the PICO principle.

### 2.3 Eligibility criteria

#### 2.3.1 Types of studies

RCTs assess the impact of biological interventions on cyclosporine and infliximab on people with severe ulcerative colitis refractory to steroids irrespective of publication status, language, or blinding procedure will be included. We will consider the inclusion of non-RCTs that report on eligibility outcomes.

#### 2.3.2 Types of participants

Participants who meet the following inclusion Truelove and Witts’ criteria will be included.^[14]^

#### 2.3.3 Types of interventions

Studies compare any form of cyclosporine to infliximab as an active therapy for severe ulcerative colitis refractory to steroids will be included. Any dosage and method of consumption about 2 treatments will be included in study.

### 2.4 Outcomes

#### 2.4.1 Primary outcomes

Rates of treatment response

#### 2.4.2 Secondary outcomes

3-month colectomy rate

12-month colectomy rate

36-month colectomy rate

Serious adverse events

Mortality

### 2.5 Data collection and analysis

#### 2.5.1 Study selection

**Figure 1:**
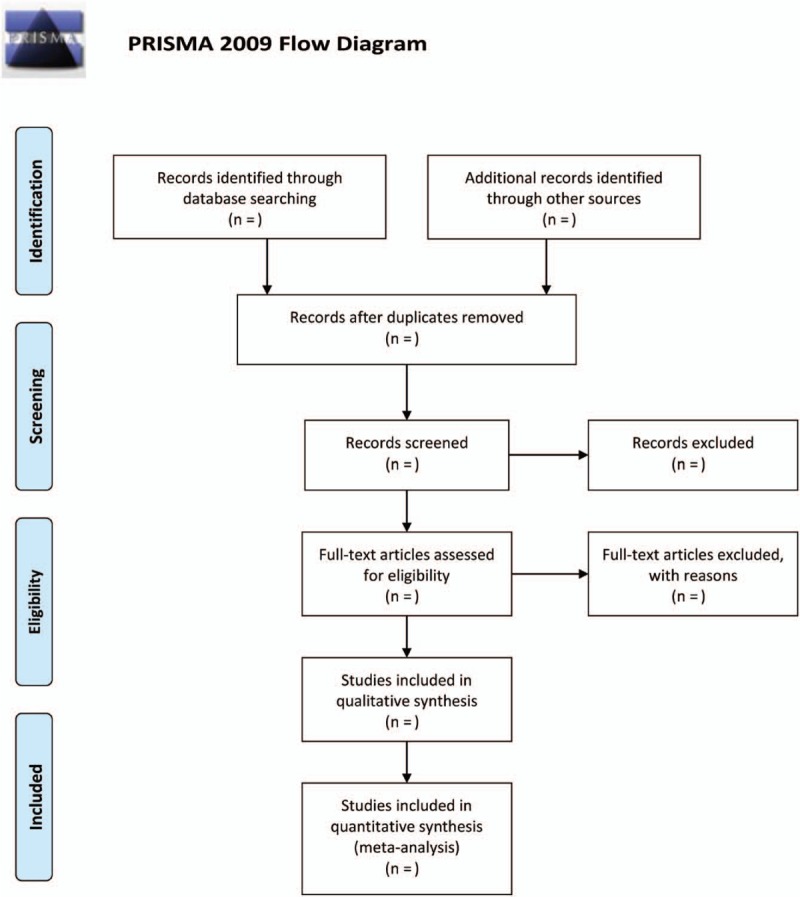
Flow diagram of selection process.

Two authors (YZ and ZC) will independently separate the result information from each investigation according to a standardized data extraction form (Fig. 1). The disagreement will be settled regarding study inclusion by a third reviewer (BZX) as necessary. In the event that information is absent or unclear, the investigation researchers will be reached for clarification.

#### 2.5.2 Data extraction and management

All the articles will be read by 2 independent reviewers, who will extract the data from the articles according to predefined criteria. The extracted data will include specific details about the authors, years of publication, study designs, sample sizes, interventions (regimens), main outcome measures, adverse effects, and authors’ conclusions. All data will be extracted and collected in a standardized spreadsheet template.

#### 2.5.3 The risk of biased assessment

Two independent reviewers (YZ and ZC) will separately survey methodological quality utilizing the Cochrane risk of bias tool.^[15]^ The conflicts cannot be settled in the review will search consensus for a third author (BZX) as required. Domains need to be evaluated will include:

1. Random sequence generation;
2. Allocation sequence concealment;
3. Blinding of participants, personal and outcome assessors;
4. Incomplete outcome data;
5. Selective outcome reporting; and
6. Other potential sources of bias.

Every class will be assessed as low, high, or vague danger of inclination and justification for judgment will be given in the attributes of included investigations segment of the survey.

#### 2.5.4 GRADE analysis

The quality of the review will be assessed utilizing the GRADE analysis.^[15,16]^ Utilizing this approach, result will be appraised as high, moderate, low, and very low quality. The degree from randomized controlled trials starts as top grade, however can be downsized in light of a few criteria

#### 2.5.5 Data synthesis

The information of review displayed as consistent variable will be utilized to perform meta-analysis to acquire the standardized mean difference (SMD), and 95% confidence interval (CI). For binary outcomes, we will figure a standard estimation of the risk ratio (RR) and its 95% confidence interval (CI). We utilized Cochran's *Q*-measurement and chi-squared test to test for heterogeneity among the included examinations. On the off chance that an *I*-squared test will be more noteworthy than half, or a *P* value of the *Q*-test will be under 0.05, demonstrating maximal heterogeneity among the included examinations, a random-effect model will be put into utilization. We will apply Begg's and Egger's funnel plot to distinguish the publication biases in the included studies.

#### 2.5.6 Dealing with missing data

We will analyze the data on an intention-to-treat basis, where by missing information without any clarifications will be thought to be treatment disappointments. We will lead an affectability examination to survey the effect of this suspicion on the impact evaluate. In the event that conceivable, we will attribute missing standard deviations. We will lead an accessible case investigation for missing ceaseless results.

#### 2.5.7 Assessment of heterogeneity

We will particularly look at the level of heterogeneity by watching the effects of the *I*^2^ measurement.^[17]^ We will undertake the synthesis of studies for heterogeneity according to guideline gave in the Cochrane Handbook to Systematic Reviews of Interventions:^[15]^

- 0% to 40%: may not be important;
- 30% to 60%: may represent moderate heterogeneity∗;
- 50% to 90%: may represent substantial heterogeneity∗;
- 75% to 100%: considerable heterogeneity∗.

#### 2.5.8 Assessment of reporting biases

In order to evaluate the production bias, we will perform funnel plots and calculate Egger's regression block for studies that report therapeutic reaction; If the *P* value is >.05, we think there is no publication bias.

#### 2.5.9 Analysis of subgroups or subsets

Data permitting, we will perform subgroup analyses to induce substantial heterogeneity. We will stratify the subgroup by different subdomains (e.g., study design, age, sex, comorbidity). If there are not sufficiently homogeneous in terms of studies, potential subgroup analyses domain will include study design.

### 2.6 Patient and Public Involvement

There are no patients or public participation in this study.

## 3 Discussion

This review will collect the most recent studies on both cyclosporine and infliximab for severe ulcerative colitis refractory to steroids and provides the evidence for clinical treatment of patients with ASUC, particularly with regard to long-term outcomes. The results of this report will be disseminated after peer review and publication.

## Author contributions

**Conceptualization:** Dongjie Wu, Zhen Yang.

**Data curation:** Dongjie Wu, Chen Zhao.

**Formal analysis:** Dongjie Wu.

**Methodology:** Liang Yao.

**Writing – original draft:** Dongjie Wu.

**Writing – review & editing:** Dongjie Wu.

Dongjie Wu orcid: 0000-0001-5760-5615
